# The Practice of Detecting Potential Cosmic Rays Using CMOS Cameras: Hardware and Algorithms

**DOI:** 10.3390/s23104858

**Published:** 2023-05-18

**Authors:** Tomasz Hachaj, Marcin Piekarczyk

**Affiliations:** Faculty of Electrical Engineering, Automatics, Computer Science and Biomedical Engineering, AGH University of Krakow, Al. Mickiewicza 30, 30-059 Krakow, Poland; mpiekarczyk@agh.edu.pl

**Keywords:** cosmic ray detection, CMOS sensors, low-power devices, image processing

## Abstract

In this paper, we discuss a practice of potential cosmic ray detection using off-the-shelves CMOS cameras. We discuss and presents the limitations of up-to-date hardware and software approaches to this task. We also present a hardware solution that we made for long-term testing of algorithms for potential cosmic ray detection. We have also proposed, implemented and tested a novel algorithm that enables real-time processing of image frames acquired by CMOS cameras in order to detect tracks of potential particles. We have compared our results with already published results and obtained acceptable results overcoming some limitation of already existing algorithms. Both source codes and data are available to download.

## 1. Introduction

In recent years, there have been many publications on the detection and interpretation of potential cosmic ray particles detected by CMOS sensors [1,2,3,4,5,6,7,8,9,10,11,12,13,14]. These papers are usually produced as part of the work of scientific teams that conduct large, international scientific projects, often based on the citizen science paradigm (CS). In a nutshell CS is one manifestation of amateur researchers or science or technology enthusiasts engaging in scientific research [15,16]. Through the use of appropriate computer systems, it is possible to integrate data collected and processed by individuals around the world.

### 1.1. Cosmic Ray Particle Detection

The observation and study of cosmic radiation is important in many scientific fields as diverse as cosmology, astrophysics, electronics and human health safety. In particular, high-energy particles and the study of their sources are of great scientific interest. Accordingly, various research efforts are being undertaken to observe and analyse such objects and phenomena. Observations can be carried out directly in space [17] or indirectly based on detectors placed on the Earth’s surface [18]. In the second case, particles or groups of particles that result from the collision of primary high-energy cosmic particles with the atmosphere are detected. Examples of infrastructure designed to analyse the effects of such interactions include large-scale stationary observatories such as the Pierre Auger Observatory in Argentina [19], IceCube in Antarctica [20] and Baikal-GVD at Lake Baikal in Russia [21,22]. Despite the large range of such research stations, they still effectively cover a relatively small area compared to the available surface of the Earth.

The concept of creating a global cloud or network of small-scale observatories is based on the collection of data from detectors scattered around the Earth. An example of this approach are scientific projects such as CRAYFIS [2], DECO [23] and CREDO [24]. Such a structure should theoretically be capable of recording and studying extensive cosmic air showers, or cascades of millions of particles reaching the Earth’s surface. Such cascades can result from the collision of even a single particle of cosmic radiation (so-called primary radiation) with particles in the Earth’s atmosphere. With detectors deployed on an Earth-wide scale, it would be possible to study the actual physical extent and energy of the particle stream, and thus consequently obtain information about the primary particle that collided in the Earth’s atmosphere. In order to try to exploit the potential available in CS, inexpensive, commonly used in everyday life and easily adaptable devices that could be turned into detectors are needed.

### 1.2. State-of-the-Art Research Using Off-the-Shelf CMOS Sensors for Cosmic Rays Detection

Low-cost CMOS cameras can be considered as a potential detector for various types of particles including cosmic ray muons [25]. Scientific papers developed under the topic of using off-the-shelf CMOS sensors for cosmic ray detection can be divided into several groups. Some of them describe large, global scientific projects in which a global network of devices is being built in the form of a distributed space observatory. Often this research is carried out in the citizen science paradigm. Such projects include The Cosmic-Ray Extremely Distributed Observatory (CREDO) [24,26], Distributed Electronic Cosmic-ray Observatory (DECO) [23,27], the Cosmic Ray Observatory Project (CROP) [28] or Cosmic Rays Found in Smartphones (CRAYFIS) [2,3]. These research groups and independent researchers publish many papers devoted to cosmic rays density estimation [29], trajectory reconstruction [30] and trace evaluation for particles classification purposed [8,14]. Published research is also devoted to methods for detecting the fact of particle impact in an off-the-shelf CMOS sensor. The use of smartphones cameras [31,32,33] or Raspberry Pi cameras [10,34] is described. The literature also includes papers on the use of CCD sensors with long duration exposures for cosmic ray detection [1] or the use of CMOS cameras for detection of interstellar meteoroids [35]. A separate topic is making observations of these particles from space [36,37] or using other modalities, for example measurements of the fluorescence light induced by air showers [38].

Very rarely publications are devoted to the topic of algorithms that are used in practice to detect the impact of a particle of potential radiation on a CMOS sensor. It is assumed that a particle hitting the CMOS detector becomes visible as a short-lived flare on the matrix of the corresponding shape. This is due to the fact that in practice it is difficult to prove that the flare is caused by the actual particle impact. In our opinion, it is worth filling this gap by discussing the algorithms used and describing their advantages and disadvantages.

### 1.3. Novelty of This Paper

Based on the literature discussed above, it can be concluded that thanks to the wide availability of relatively cheap and mobile CMOS cameras, the issue of particle detection with their help is a very current research topic [27]. Often such solutions are used in citizen science projects, in which participants use their own off-the-shelf equipment and dedicated software to participate in global scientific projects. The discussion of software design and preparation of low-power consumption hardware and CMOS sensors for the detection of potential cosmic radiation, along with evaluation of its performance, presented in this paper, is the main novelty of this work. We discuss and presents the limitations of up-to-date hardware and software approaches to this task. Our solutions overcome some of these limitations being in a real-time algorithm processing image frames acquired by CMOS cameras in order to detect tracks of potential particles.

## 2. Materials and Methods

### 2.1. Cosmic Ray Particles Detection Using CMOS Sensors

The procedure for using CMOS-type imaging sensors to record and detect the movement of high-energy particles requires careful obscuring of the camera. The lack of exposure of the sensor to visible light provides the opportunity to observe penetrating particles. The image thus recorded may contain a potential particle track. In this case, it should mostly consist of black pixels due to the full obscuration of the matrix. When the obscuration condition is met, if a particle of primary or secondary cosmic radiation, such as protons or muons or possibly a particle of a local radiation source, passes through the active layer of the CMOS camera, it will excite some of the pixels located in the homogeneous area. A few to a few dozen pixels, arranged in clusters of shapes ranging from small solid circular shapes (dots) to elongated lines (tracks), should then be significantly brighter against a more or less uniform black background. Irregular curves or twisted particle tracks usually correspond to high-energy electrons or particles excited by local radiation. The events in which either dots or long linear paths are visible could potentially be traces of cosmic ray muons that have passed through the camera at either acute or high angles [26]. The signals are roughly proportional to the ionization energy loss in the individual pixels of the array.

More precisely speaking, dots or long and straight tracks are most often caused by high-energy (minimally ionizing, ~GeV) cosmic ray particles. These can be secondary particles (especially muons) observed at sea level or possibly primary particles (especially protons), but these usually occur at high altitudes, such as the cruising altitude of commercial airliners (~10 km). Worms represent traces of low-energy (~MeV) electrons arising from radioactive decays in or around the phone material [27].

### 2.2. Hardware Requirements

The basic requirement that a CMOS camera must meet in order to be useful for acquiring potential cosmic radiation is:

- The ability to operate in a mode without colour interpolation based on neighbouring pixels. This is usually achieved either by setting the maximum available resolution on the camera or by downloading raw data (RAW mode). In CMOS cameras spatial down sampling may occur due to binning (averaging of neighbour pixels), or via decimation (individual pixels are selected to represent larger blocks of pixels) [31].
- The camera has to be configured to transmit uncompressed data. Many USB cameras transmit data in MJPEG format by default, rendering such images useless for post-pixel-level analysis.
- The camera should download data continuously, thus maximising the observation time [1]. This is accomplished in practice by running the data transfer in video mode rather than post-editing frames. This unfortunately results in reduced data resolution.

A significant problem when establishing a connection to a CMOS sensor is the functions and configuration parameters provided by the driver. Based on our experience working on the CREDO project, we noticed that many off-the-shelf CMOS USB cameras have compression of transmitted frames set by default, and due to the lack of an available driver, it is not possible to access the uncompressed data. Furthermore, a number of modern smartphones use advanced filters that remove high-frequency noise, thus virtually levelling the effect of cosmic ray registration. These facts are, in our opinion, the greatest difficulty to be overcome when preparing CMOS hardware for the detection of potential cosmic radiation. Low-level driver programming for the circuits is out of the scope of this paper, but we mention it because some CMOS cameras will be impossible to use in practice without such knowledge, and it is worth checking this first.

Another important aspect is the selection of a suitable computational platform for pre-processing the captured images to detect the presence of potential cosmic radiation. Of course, if we use a modern PC-class computer the calculations will not pose any problems. However, since data recording is a lengthy process we are eager to minimise the electricity consumed. For this reason, it is advisable to use microcomputers or smartphones that have low power consumption. However, if we use microcomputers, image processing with full HD resolution (1920 × 1080 pixels) poses a serious computational problem due to the limited computing capacity of the processor. When designing algorithms for this type of hardware, it is necessary to reduce the need for repeated iteration over the entire image resolution, as well as to use GPU support or multithreading where possible. It is also possible to use techniques that allow reduction in resolution without loss of relevant information, such as pooling, known from deep neural networks.

### 2.3. Proposed Hardware for the Long-Term Test of Potential Cosmic Ray Detection Algorithms

**Figure 1:**
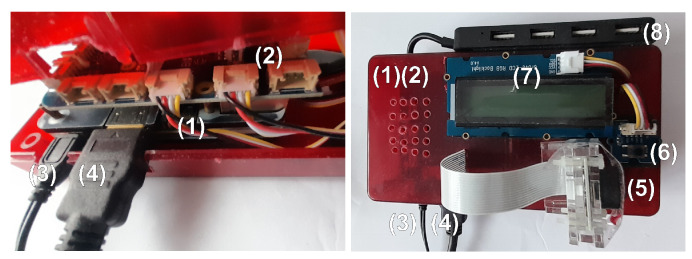
This figure present a low-power device developed to perform the long-term test of potential cosmic ray detection algorithms. On the left is the interior of the casing, and on the right a top view of the device. (1) Raspberry Pi 3 microcomputer; (2) GrovePi+ hat; (3) power source (5.1 V, 2.5 A); (4) HDMI; (5) Raspberry Pi camera; (6) button to light-up LCD display; (7) LCD display that shows processor temperature and hit count; (8) USB hub connected to the Raspberry Pi.

We have developed the hardware for the long-term test of potential cosmic ray detection algorithms with low-power energy consumption, see Figure 1. We used the Raspberry Pi 3 microcomputer with 1.2 MHz processor and 1 GB RAM. We installed the Raspberry Pi operating system. The image processing algorithms use the OpenCV library [39] as the backbone. For test and validation purposes we utilised two CMOS Raspberry Pi camera versions. Camera version 1.3 utilises the OV5647 matrix. Camera version 2 utilises the IMX219 matrix. We used these cameras interchangeably. Earlier research [34] showed the suitability of CMOS hardware for cosmic ray detection and measurement.

We used the OpenCV VideoCapture module to acquire images. Cameras resolution was set to 1920 × 1080. This is the highest resolution we could obtain using this hardware–software setup and available drivers without using video stream compression. We used the C++ OpenCV API instead of Python API to speed up calculations.

### 2.4. State-of-the-Art Algorithms for Potential Cosmic Rays Detection

Cosmic rays acquired at ground level are relativistic-charged particles. This means cosmic rays penetrate through the sensor depositing minimum ionization energy loss, leaving trajectories with small dots or straight lines [10]. We cannot, however, precisely calculate the brightness of these traces. Due to this it is difficult to distinguish between background and actual cosmic rays.

Virtually all algorithms for detecting potential cosmic rays boil down to performing per-pixel thresholding on a newly acquired image. However, there are a number of factors that must be taken into account:

- noises generated by the camera, including hot pixels (pixels that do not react linearly to incident light [40,41]);
- the unknown limit above which we are dealing with potential cosmic ray;
- light recorded by the sensor due to inaccurate shielding of the camera lens.

The algorithms used in practice solve the above problems differently. These are usually heuristics whose adaptive parameters have been determined experimentally or are calculated adaptively during operation. In the following subsections we will discuss these types of approaches.

#### 2.4.1. Single Fixed-Threshold Methods

The simplest approach for detecting potential particles is to use simple thresholding. Images from a CMOS camera that contain pixel values above a certain threshold will be counted as potential cosmic rays [2,3,10]. Published papers rarely provide the exact value of such a threshold or how it was estimated.

In [8,33], the threshold was determined by a calibration procedure. During it the dark noise was measured and the bright threshold was obtained (default: 3 times the average noise but not less than 80 and not higher than 160). Furthermore, [32] used an initial calibration procedure to estimate the threshold. The approach described in [31] used a two-level trigger system for potential cosmic ray detection. The first trigger examines each frame, rejecting those which have no clean pixels above a given threshold. The second threshold examines each pixel, storing those which have luminance above a second threshold and their neighbours. The choice of threshold is performed by the on-device software to achieve a remotely configurable frame pass rate. The choice of threshold is also optimise to obtain the frame acquiring rate of 0.33 Hz.

Algorithm 1 is a pseudocode of the CREDO algorithm [33] for potential cosmic ray detection for mobile devices which is a good representation of a single fixed threshold method.
**Algorithm 1:** CREDO algorithm [33] for potential cosmic ray detection for mobile devices
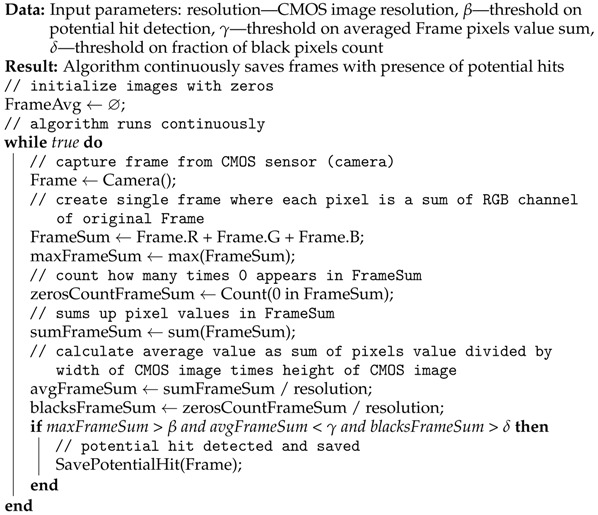


    As can be seen in Algorithm 1, the algorithm repeatedly acquires data from CMOS sensors and examines three conditions. At first it checks if the maximal pixel value is above a certain threshold β. This condition checks the presence of the potential particle hitting the CMOS matrix. The second condition checks if the average pixel value is below threshold γ. This is performed in order to examine if the overall brightness of the image is not too high—this situation happens if the camera is not correctly covered. The third condition checks if the number of black pixels are above threshold δ. This condition is somehow redundant with previous one. The default values of threshold parameters are β = 120, γ = 40 and δ = 0.04.

#### 2.4.2. Adaptive Threshold Methods

Thomas C. Andersen (Research Director at NSCIR.ca, https://nscir.ca, accessed on 14 May 2023) proposed an approach that utilises the moving average and sophisticated division of the image into subregions to improve ray detection stability and robustness. His algorithm is now hosted in the CREDO repository https://github.com/credo-science/credo-cosmic-ray-detector-ios (accessed on 14 May 2023). The pseudocode of Thomas C. Andersen’s algorithm is presented in Algorithm 2.
**Algorithm 2:** Thomas C. Andersen algorithm for potential cosmic ray detection for mobile devices
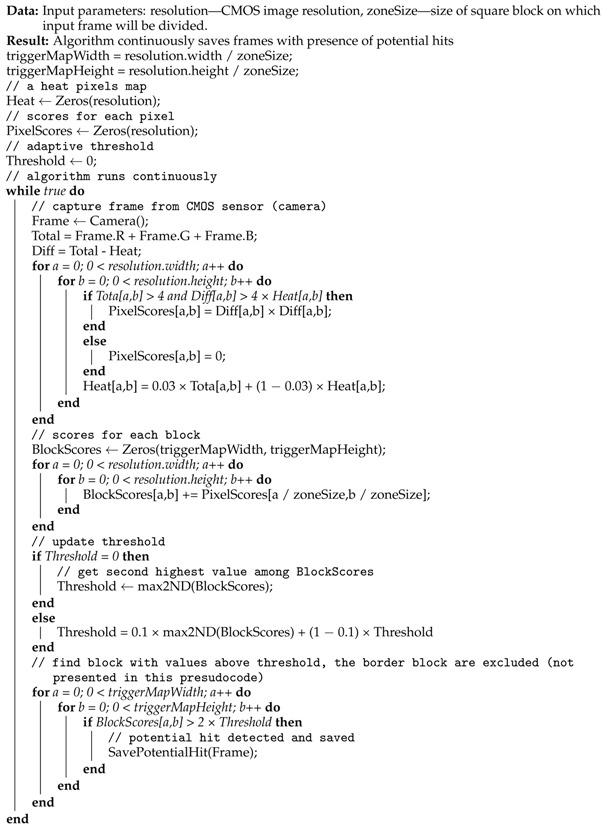


    Algorithm 2 loops through all pixels of the frame in order to calculate the difference between the actual and previous intensity of the frame’s pixels. The adaptive moving average of each pixel is stored in an array the same size as the camera frame (Heat). This is used to detect hot pixels. To each pixel a scoring is assigned (PixelScore). Then the algorithm splits the frame into multiple square subregions (blocks) with size equal to zoneSize × zoneSize (the default zoneSize is 20). A score is calculated for each block, which is the sum of each pixel score contained in the block. Then the score value for each block is examine to verify if it is above double the second threshold (Threshold). The threshold is calculated as a moving average of the second higher value of the block score.

Most per-pixel operations can be rewritten to be used in single-instruction multiple-data parallel processing on GPU. Andersen did so in his implementation.

### 2.5. Prototype Algorithm for a Low-Power Environment for Long-Term and Continuous Potential Cosmic Ray Detection

**Figure 2:**
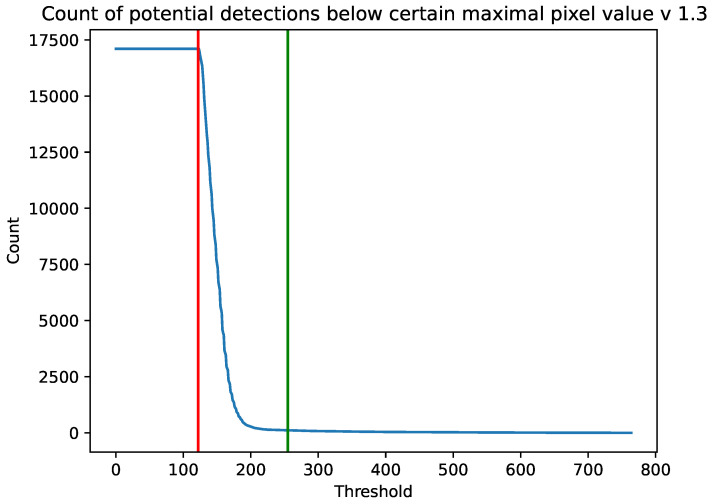
Number of potential detections with a maximum pixel value below a certain value. Results are accumulated for the whole experiment on a Raspberry Pi camera 1.3. The vertical red line is a minimum value of θ=122 from Algorithm 3 from which we acquired data. The green vertical line is θ=255.

**Figure 3:**
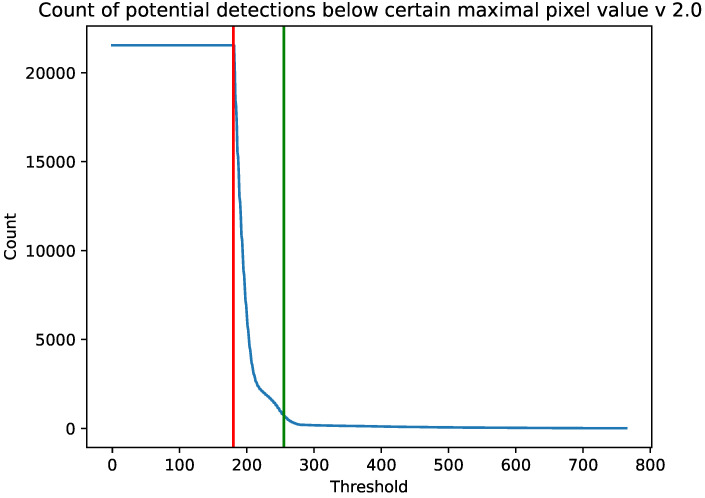
Number of potential detections with a maximum pixel value below a certain value. Results are accumulated for the whole experiment on a Raspberry Pi camera 1.3. The vertical red line is a minimum value of θ=180 from Algorithm 3 from which we acquired data. The green vertical line is θ=255.

Algorithm 2 is far more complicated than the single fixed threshold approaches represented by Algorithm 1. It uses two adaptive thresholds to exclude local camera noises and determine the value at which a block should be classify as one that contains a hit. It has however several drawbacks:

- It has several loops over the whole image resolution. Without GPU acceleration, which is not always available, the algorithm runs slower on low-power devices, such as smartphones and microcomputers.
- Using fixed-sized blocks with diameter of about 20×20 is a huge reduction in image resolution. In practice, the number of pixels is reduced 400 times. Furthermore, the fixed spatial position of the blocks might disturb the continuity of events, especially when events are registered at the border between blocks and split between two or even four block. In this case the block score might be below the threshold.
- As will be shown later in Section 3, the moving threshold based on the block score might generate over-detection of potential hits. This is due to the fact that CMOS sensors might be affected by random relatively high-value spot-like noises that, due to their frequency (much higher than expected background radiation), are not caused by cosmic rays hits (see Figure 2 and Figure 3).

Algorithm 3 aims to overcome the above issues by a different approach to dimensional reduction and continuous averaging.

Algorithm 1 repeatedly acquires data from the CMOS sensors. In order to reduce the computational complexity, a max pooling operation with size 2 is performed on the acquired frame and the resulting image is kept in the variable FramePool. Max pooling is equivalent to block aggregation in Algorithm 2; however, it has a higher resolution and does not average the pixel in the block. Max pooling keeps information about hot pixels and potential cosmic ray hit events. The results of the max pooling are averaged by Gaussian blur and kept in a new variable FrameGaussian. Gausian blur removes local low-value noises keeping the high-value pixels that might contain potential cosmic ray hits. Furthermore, applying Gaussian blurring creates a potential hot pixel map, similar to Heat in Algorithm 2. Our algorithm calculates a pixel-level adaptive threshold for potential hit detection based on a moving average that is kept in the variable FrameAvg. First, imc iterations (default 100) of the algorithm are used to calculate the initial threshold. The detection of potential hits happens when the maximal value of the FramePool in a given pixel is four times higher than the maximal value of the FrameAvg (compared with Algorithm 2) and the max FramePool value is above the given threshold θ.
**Algorithm 3:** Our proposed algorithm for potential cosmic ray detection designed for low-power consumption microcomputers.
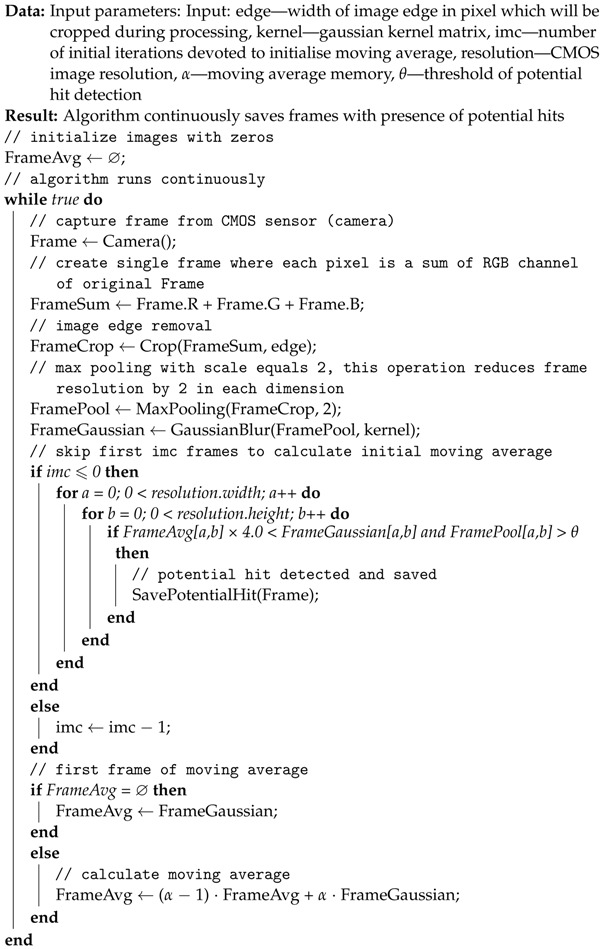


## 3. Results

We implemented our algorithm using a Raspberry Pi microcomputer, often used for prototyping and testing image-processing algorithms [1,10] (see Section 2.3). The resolution of the Raspberry Pi cameras was set to 1920 × 1080 (2 megapixels). The edge was set to 30 pixels, and the Gaussian kernel size was 3 × 3. The theta threshold was set to 255. The experiment for the 1.3 camera took 19 days and for the 2.0 camera took 12 days. The source codes and data from our experiments can be downloaded from https://github.com/browarsoftware/cmosdetector (accessed on 14 May 2023).

In Figure 2 and Figure 3 we present the number of potential detections with a maximum pixel value below a certain value for the cameras 1.3 and 2.0, respectively. The results are accumulated from the whole experiment. The vertical red line is a minimum value of θ=122 from Algorithm 3 from which we acquired data. The green vertical line is θ=255 which we used as the threshold for the rest of the calculations. We chose this value because, for both the 1.3 and 2.0 Raspberry Pi cameras, the number of images containing potential cosmic rays was much larger than the estimated background radiation. Above this value, on the other hand, there was a plateau.

Note that in Algorithm 2, assuming that every 100th pixel has a value of 4 (the mean pixel value for sensor 1.3 is 1.191±0.500 and for 2.0 is 1.131±0.003, see Table 1), then for a 20×20 block size the block score from which the acceptance threshold is counted will be $4^{2}\times 20^{2}\times 0.01=64$. Therefore, Algorithm 2 has a large over-detection.

**Figure 4:**
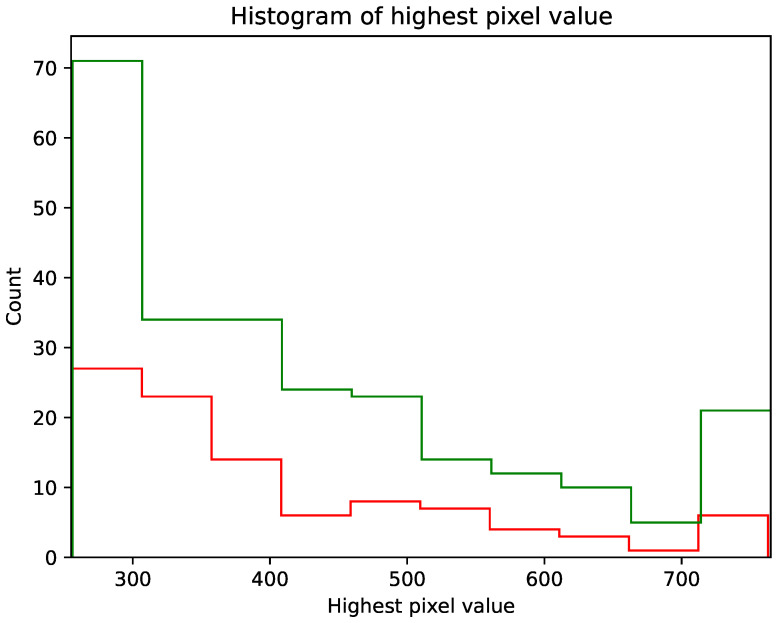
Histogram plot showing the frequency of the given maximum pixel value on the potential hit images. The green histogram is data from the Raspberry Pi camera 2.0 sensor and the red histogram is data from the Raspberry Pi camera 1.3 sensor.

**Figure 5:**
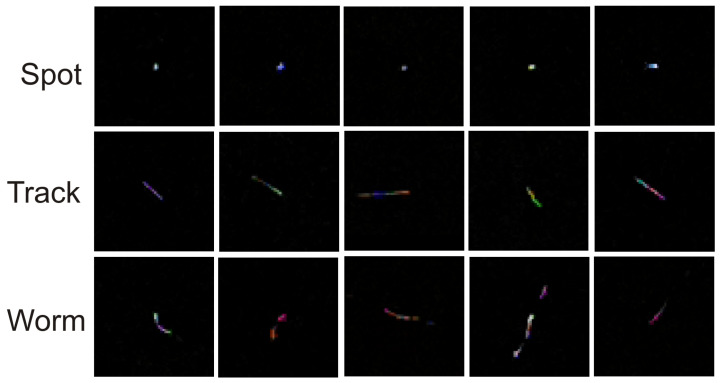
Example potential hit images acquired using Algorithm 3 with the 1.3 sensor classified by their shapes. Each image is cropped to a default resolution of 60 × 60.

**Figure 6:**
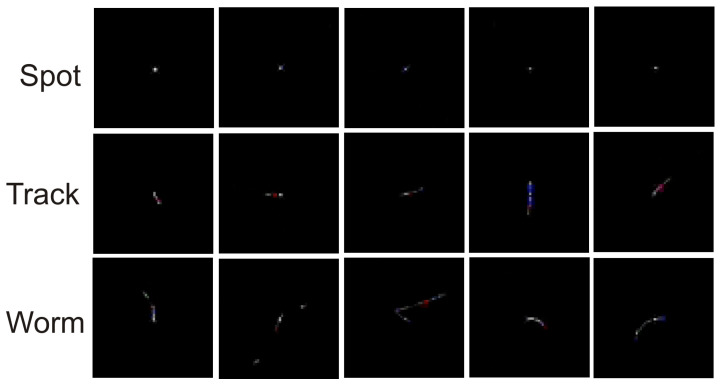
Example potential hit images acquired using Algorithm 3 with the 2.0 sensor classified by their shapes. Each image is cropped to a default resolution of 60 × 60.

Throughout the duration of the experiment using Algorithm 3, 99 potential particles were collected using sensor 1.3 and 248 potential particles were collected using sensor 2.0. Figure 4 shows the frequency of the set maximum pixel value on the potential hit image. The minimum value on the X-axis was 255 (value) and the maximum value was 765. Figure 5 and Figure 6 show example potential hit images acquired using Algorithm 3 with the 1.3 sensor and 2.0 sensors, respectively. Images were classified by their shapes as spots, tracks and worms. Each image was cropped to a default resolution of 60 × 60, where the centre point was the pixel with the highest pixel value.

In Table 1, we present the number of captured potential cosmic ray images of different classes through the Raspberry Pi cameras with the 1.3 and 2.0 sensors. The results are compared with data reported in the literature [34,42].

## 4. Discussion

As can be seen in Figure 2 and Figure 3, versions 1.3 and 2.0 of the sensors have different sensitivities. Although the shapes of the graphs depicting the number of potential detections with a maximum pixel value below a threshold are similar, sensor 1.3 reaches a plateau earlier than sensor 2.0. As can also be seen from the histogram in Figure 4, each stratified range of the maximum pixel value sensor 2.0 counted more potential particles than sensor 1.3.

The example potential hit images presented in Figure 5 and Figure 6 are very close to those presented in state-of-the-art papers, and classes of shapes from each of the defined classes (spots, tracks and worms) can be found among them.

**Figure 7:**
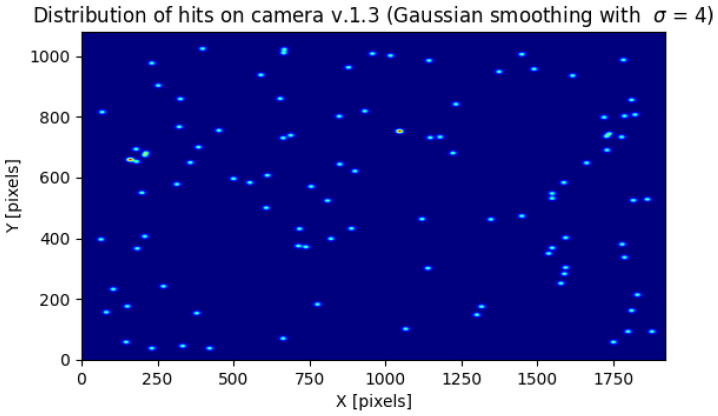
The distribution on the sensor array of 1.3 potential cosmic ray hits. To increase the visibility the image was smoothed with a Gaussian filter with σ=4.

**Figure 8:**
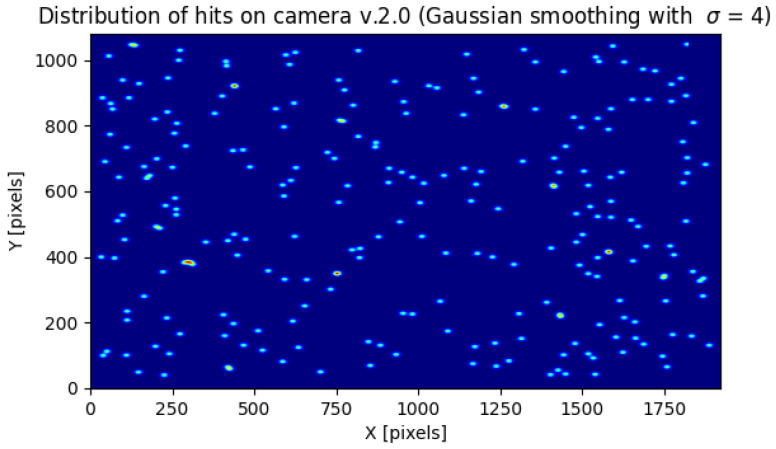
The distribution on the sensor array of 1.3 potential cosmic ray hits. To increase the visibility the image was smoothed with a Gaussian filter with σ=4.

**Figure 9:**
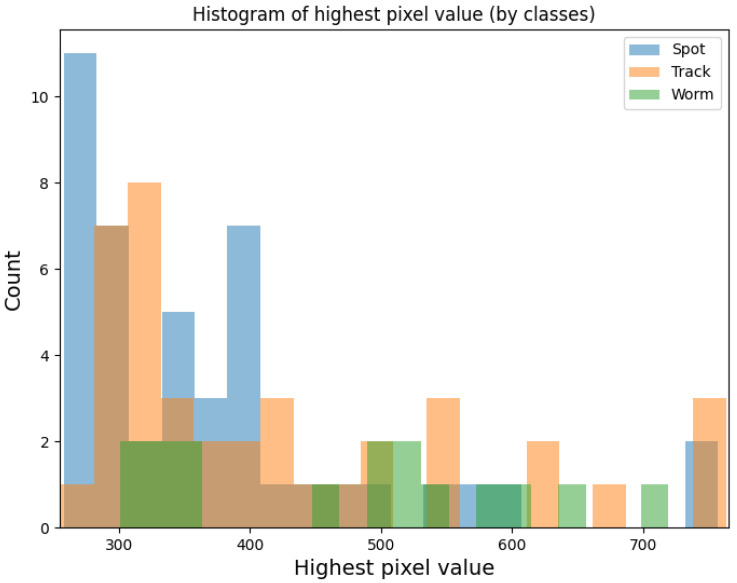
Histogram plot for the 1.3 sensor array showing the frequency of the set maximum pixel values for the potential hit image grouped according to image classes.

**Figure 10:**
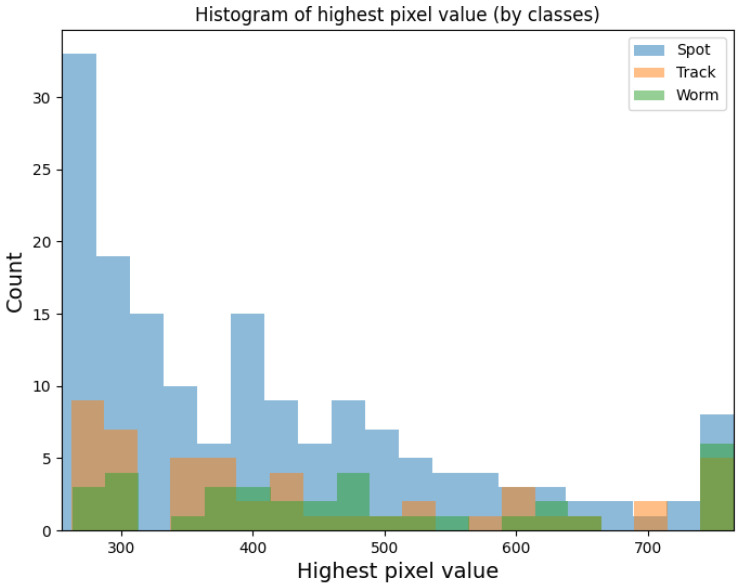
Histogram plot for the 2.0 sensor array showing the frequency of the set maximum pixel values for the potential hit image grouped according to image classes.

As can be seen in Figure 7 and Figure 8 the distribution of potential particles on the matrix does not show regularity. There are also no pixels excited more than once during the entire experiment. Therefore, this suggets the distribution of the detected potential particles was random during the experiment. This means that either the hot pixels did not occur or were they eliminated by Algorithm 3. As the histograms in Figure 9 and Figure 10 indicate, the sensors often register spots followed by tracks and worms. In the case of sensor 2.0, however, spots are much more numerous than the other object classes. Tracks and worms were spread over the entire range of maximum pixel values with no regularity.

There are almost no scientific papers describing the characterized measurable values of potential cosmic rays, i.e., the maximum pixel brightness obtained with off-the-shelf CMOS cameras and what frequency such equipment captures potential cosmic rays. This is due to the varying sensitivity characteristics of the camera arrays and the different detection algorithms. However, according to [42,43], the muon flux density at the Earth’s surface is about $1\frac{\mathrm{muon}}{{\mathrm{cm}}^{2}\cdot \min}$. Therefore, we can estimate that over a period of 1 h, 1 mm${}^{2}$ of the Earth’s surface averages $\rho =0.6\frac{\mathrm{muon}}{{\mathrm{mm}}^{2}\cdot h}$. Ref. [34] designed four Raspberry Pi 2.0 cameras placed in a vertical stack detector at ~1180 h, and registered 78 candidate events across all 4 sensors. That is, $f=0.066\cdot \frac{\mathrm{muon}}{h}$ and an estimated $\rho =0.007\cdot \frac{\mathrm{muon}}{{\mathrm{mm}}^{2}\cdot h}$. We presented a comparison of these results and those obtained by us in Table 1. The solution registered significantly more events than in [34]; however, it should be noted that a stack of four detectors was used, only registering simultaneous events. Therefore, it is natural that [34] recorded fewer events. In the case of the estimated results in [34], sensor 1.3 detected 34 times fewer events than the expected background radiation, and sensor 2.0 detected 8 times fewer events than the expected background radiation.

Since it cannot be assumed that the CMOS sensor camera is capable of detecting 100% of the radiation, one could try to improve this result by tuning θ of Algorithm 3. Note, however, that as θ decreases, the number of visible detections in Figure 2 and Figure 3 increases exponentially. Since this increase is not caused by the action of an adaptive threshold, whose role is to remove hot pixels, the selection of an appropriate threshold is crucial here.

In fact, a dynamic threshold only subtracts hot pixels, and is not a good threshold for potential cosmic ray detection because it has an inadequate filtration threshold. This is because the average pixel values recorded by the obscured camera is relatively low (see Table 1). This means that if the acceptance threshold of potential cosmic rays was only based on the value calculated from the average pixel, the algorithm would have a false acceptance rate that was too high and the estimated ρ would greatly exceed the value in [42]. We have already initially discussed this using an example of Algorithm 2 in Section 4. The use of a global threshold θ, as used in our algorithm, is therefore highly recommended.

## 5. Conclusions

The algorithm proposed in this paper for detecting potential cosmic radiation has proven to be an effective solution offering particle capture with acceptable ρ. In practice, any CMOS sensor must be calibrated to establish an appropriate θ threshold before using it as an effective measurement device. An adaptive threshold can be used in practice to eliminate hot pixels. Assessing the minimum acceptable value of pixel brightness above which we are dealing with a potential cosmic ray is in principle always based on heuristics, the threshold of which must be defined experimentally.

It should be noted that our work is one of the few available studies in which we present a complete algorithm for this image modality and its long-term evaluation. Our proposed method combines the positive features of the algorithms proposed here and described in the literature, such as adaptive hot pixel removal threshold (not at the region level) and relatively high computational speeds without using GPU support.

We anticipate that our proposed algorithm performs well as a tool for CS based projects such as CREDO. In the future, it would be advisable to conduct validation studies on various types of modern smartphone cameras. This poses some technical challenges due to the various low-level APIs that are on these devices.

## Figures and Tables

**Table 1:** Comparison of the number of captured potential cosmic ray images of different classes through the 1.3 and 2.0 Raspberry Pi camera sensors. The mean pixel value is the averaged pixel value in the image ± standard deviation, f is the detection frequency of potential cosmic rays, area is the area of the CMOS sensor, and the estimated ρ is the calculated density of background radiation. The results are compared with data from the literature [34,42,43]. We excluded #Worms from calculation.

Sensor Model	Mean Pixel Value	Acquisition Time (h)	#Spots	#Tracks	#Worms	#Total (Excluding #Worms)	f ($\frac{\mathrm{muon}}{h}$)	Area (mm${}^{2}$)	Estimated ρ ($\frac{\mathrm{muon}}{{\mathrm{mm}}^{2}\cdot h}$)
1.3 (our research)	1.191±0.500	456	44	39	16	83	0.182	10.302	0.018
2.0 (our research)	1.131±0.003	288	163	50	35	213	0.740	10.156	0.073
2.0 (stack of four [34])	-	1180	-	-	-	78	0.66	10.156	0.007
Reference level [42,43]	-	1	-	-	-	-	0.6	1	0.6

## Data Availability

Source codes can be downloaded from: https://github.com/browarsoftware/cmosdetector accessed on 8 March 2023.
